# On the mechanism of NPM1 mutations in acute myeloid leukemia

**DOI:** 10.1038/s41375-025-02722-3

**Published:** 2025-07-28

**Authors:** Michael R. Lieber, Chih-Lin Hsieh

**Affiliations:** 1 USC Norris Comprehensive Cancer Center and Departments of Pathology, Molecular Microbiology & Immunology, Cancer Biology, and Section of Molecular & Computational Biology (Department of Biological Sciences), Los Angeles, CA USA; 2 USC Norris Comprehensive Cancer Center and Department of Urology, University of Southern California, Los Angeles, CA USA

**Keywords:** Diseases, Acute myeloid leukaemia, Biochemistry

Molecular assessment of *NPM1* gene alterations is critical for prognosis and therapeutic decisions in AML [1–4]. *NPM1* mutations occur in ~35% of AML patients. Compilations of literature cases illustrate the consistent location in the last exon of the *NPM1* gene [5]. These mutations alter the subcellular localization of NPM1 protein, affecting key processes.

A 4-nucleotide (nt) insertion is observed in nearly all (2321 out of 2322 total events) *NPM1* gene mutations in human AML [5]. This insertion occurs at a specific phosphodiester bond in 97.2% (2257 events out of 2322 total) of cases, with the 4 nt sequence 5’-TCTG-3’ added in 1750 cases (75.4%); the 4-nt sequence 5’ xxTG 3’ (xx designates any nucleotides) added in 414 cases (17.9%); and other 4-nt sequences added in 93 cases (4%). Slippage during DNA synthesis has commonly been assumed as a possible mechanism for all these events. However, the variability of the DNA sequence of the insertions is inconsistent with simple slippage, forcing consideration of a more complex mechanism [5].

One group suggested addition by TdT during DNA replication slippage for all NPM1 gene insertions [5]. But under physiologic conditions, TdT typically requires a broken double-stranded DNA end at which to act, and slippage during replication does not involve a double-stranded DNA end [6]. Also, TdT nt additions vary broadly in sequence and length, in contrast to the predominant TCTG addition immediately downstream of the existing identical TCTG sequence at that location. Most importantly, TdT is not expressed in myeloid precursors, myeloid cells, or the very large majority of AML tumor cells. All these factors weigh heavily against the involvement of TdT.

*A key question in considering any NPM1 mutation mechanism is: what causes the DNA lesion that results in the 4-nt nucleotide insertion in 97.2% of cases at the same nt location?* The sequence of the 4-nt insertions leads to the same or a highly similar leucine-rich nuclear export sequence [7]. The predominant 75.4% mutation with TCTG addition and two less frequent variants with CCTG (6.5%) and CTTG (1.5%) additions have the same amino acid changes (DLCLAVEEVSLRK). Therefore, the observation of a predominant TCTG insertion indicates more than functional selection. This marked predominance is most consistent with mechanistic features of a DNA repair process. The *NPM1* events rarely show nucleotide loss; and this argues against a double-strand break because these almost always involve nt loss (along with variable nt addition) [8].

Few processes in human cells incise DNA with the locational precision of the NPM1 mutation. Apobec3 enzymes are constitutively expressed widely among human cells for protection against viral infection, but a significant level of off-target mutation by Apobec3 enzymes is documented in genome-wide sequencing studies of healthy human cells [9]. A 5’-C**C**-3’ sequence on the bottom strand at the site of the insertions in the *NPM1* gene corresponds to the *optimal target* of the cytidine deaminase enzyme, Apobec3G, which would convert the bold C to a U to create a U:G mismatch (Fig. 1 and Supplementary text) [10–12]. This DNA damage would trigger base excision repair involving uracil glycosylase (UNG2) and apurinic/apyrimidinic endonuclease (APE1) to create a one nucleotide gap. Fill-in synthesis by polymerase beta would restore the sequence as it was. Mutations arising from Apobec3 deamination are well-known to occur, and the imperfect restoration may lead to the changes observed in neoplasms [12]. To arrive at the sequence outcome that is observed in the large majority of *NPM1* mutations, transient breathing adjacent to the one nt gap would permit pol beta to fill-in 4 nts beyond the one nt gap, and misincorporation errors by pol beta have been documented in such circumstances (see Supplementary text) [13–15]. Ligation of the added nts with the displaced flap would permit a second set of the same 4-nts to be incorporated downstream of the first 4-nt TCTG (Fig. 1, top strand), as is seen in 75.4% of patient mutations.

**Fig. 1:**
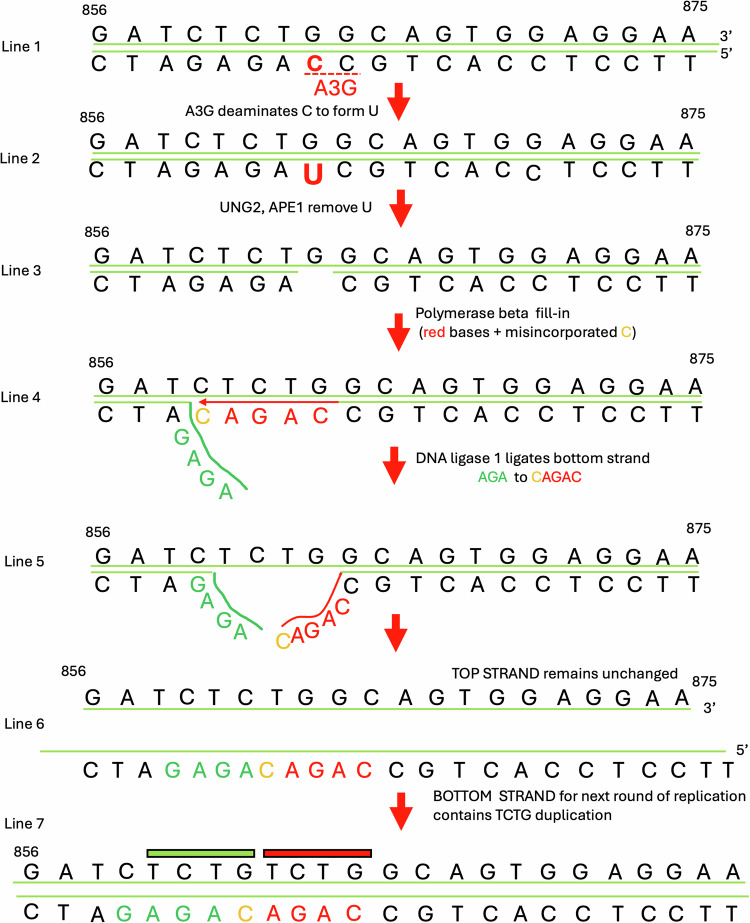
Proposed mechanism for the most frequent NPM1 mutations. For the several reasons enumerated in the Supplementary text, DNA repair synthesis must begin on the bottom strand at or to the right (downstream) of the original top strand TCTG sequence. Apobec3 enzymes are cytidine deaminases that convert C to U. Apobec3G (A3G) prefers to deaminate the bold (red) 3’ **C** in the 5’-C**C**-3’ dinucleotide motif on the bottom strand (marked by a dashed red line in LINE 1 of the figure), which is at the righthand edge of the 5’-TCTG-3’ sequence on the top strand [10, 11]. In LINE 2, deamination at this bottom strand **C** would convert it to a U. The enzymes uracil glycosylase (UNG2) and apurinic/apyrimidinic endonuclease (APE1) would convert the U to a one base gap (on the bottom strand of the duplex as in LINE3). An abasic site would usually be repaired back to its original sequence by base excision repair, involving DNA polymerase beta (pol beta). But breathing of the 5’-AGAG-3’ (bottom strand) sequence directly adjacent to the one nt gap would permit pol beta to extend beyond the one nt gap, as shown in LINE 4. In LINE 4, a 1-nt misincorporation (**orange C** nt opposite the top strand C) would generate the bottom strand sequence 5’- CAGAC-3’ [13–15]. In LINE 5, when this 5’-CAGAC-3’ is ligated to the displaced 5’-AGA-3’ flap, the sequence on the bottom strand is the most common outcome in the NPM1 gene mutation. During the next round of DNA replication, this strand would give rise to the duplication of the top strand TCTG characteristic of over 75.4% of NPM1 mutations (shown in LINE 7). The thick **green** bar above the sequence marks the location of the original sequence, and the thick red bar above the sequence marks the portion that is newly synthesized or duplicated. The numbering of nucleotides refers to the coding DNA sequence (e.g., c.856), consistent with Borrow et al. [5].

Of note, pol beta nucleotide misincorporation in gaps longer than one nt has a 60-fold higher misincorporation rate than normal, and two adjacent misincorporations are particularly elevated (Supplementary text) [13, 14]. These aspects of pol beta may lead to 6.8% of NPM1 mutation events with one nt of misincorporation (CCTG) and 8.5% of mutation events with 2-nt of misincorporation (CATG) in the second 4-nt repeat, all at the same location mentioned above. Additional, but much less frequent DNA sequence variations in the 4-nt addition, account for 6% [5].

Importantly, Apobec3G (and Apobec3F) mRNA is well documented to be expressed in AML cells and in normal human cells in the myeloid lineage, including myeloid precursors [16, 17]. This makes it more likely that Apobec3 and pol beta enzymes could account for the precise location of the strongly preferred 4-nt insertion at the NPM1 gene.

Given that NPM1 and FLT3 mutations are both common in AML, even in the same malignant clone in ~30% of cases, could FLT3 mutations occur by a related mechanism [7, 18]? FLT3 internal tandem duplications (ITD) are quite diverse, including events that initiate within its exon 14 but terminate variably within exon 14, intron 14, or exon 15. With such diversity, it is very difficult to define a single pathway of events. However, among recurring patient FLT3 duplications, the two most frequent start positions both have *optimal* Apobec3F deamination sites (Supplementary Fig. 1, bold C in the 5’ T**C**T 3’ sequence; red font in the Supplementary figure) [10–12]. A series of plausible steps for one of these is diagrammed (Supplementary Fig. 1). After an Apobec3F deamination at this site, the nick generated during repair could permit slip-mispair priming. No other causes or lesions have ever been proposed to account for such a frequently used duplication start position. In addition to this predominant Apobec3F site in FLT3, there are other Apobec3F and Apobec3G sites nearby that may contribute to a subset of the less frequent FLT3 ITD start positions.

Both NPM1 and FLT3 are highly transcribed genes in the myeloid lineage and in AML tumor cells [5, 7]. Transcription increases the likelihood of Apobec3 action at these genes due to the increase in local single-strandedness within and upstream of the transcription bubble [19]. Several Apobec3 enzymes have two cytidine deaminase domains, called CD1 and CD2. The CD2 domain is catalytically active for cytidine deamination. The CD1 domain has evolutionarily lost its deaminase catalytic capability but has retained an important function in binding single-stranded nucleic acid [19, 20]. We have proposed the RNA tether model for substrate targeting by two-domain enzymes of this family, which includes Apobec3B, 3D, 3F and 3G, and the evolutionarily related cytidine deaminase called activation-induced deaminase (AID) [21]. AID has a demonstrated role in immunoglobulin (Ig) somatic hypermutation (Ig affinity maturation) and Ig class switch recombination [22]. We showed that the CD1-like domain of AID binds ssRNA [21]. Based on this, we proposed that AID and Apobec3 enzymes retaining the CD1 domain, such as 3F and 3G, can preferentially deaminate DNA at transcribing genes by binding to the RNA arising in the vicinity of the transcription bubble; hence, the designation of RNA tethering [19]. The fact that the NPM1 and FLT3 genes are transcriptionally active in myeloid cells increases their likelihood to be targets for the abundant Apobec3F and 3 G in myeloid cells that give rise to AML.

Many valuable studies have highlighted the consistency of the specific genetic changes of NPM1 and the more diverse profile of change at FLT3 in AML. The missing element remaining has been the cause of the lesion that leads to these consistent and medically important events. If this model is proven correct, it will markedly deepen our understanding of the molecular etiology of this central leukemia of children and adults. Moreover, it would foster work to understand how factors that modulate Apobec3 expression, such as chronic immune stimulation by infection or autoimmune disease, may increase the risk of developing AML [23].

## Supplementary information

On the Mechanism of the NPM1 Mutation in Acute Myeloid Leukemia
