# Supplementary material for: On the mechanism of NPM1 mutations in acute myeloid leukemia
Source: Leukemia. 2025 Jul 28;39(10):2340–3. doi: 10.1038/s41375-025-02722-3 (PMC12463662; doi:10.1038/s41375-025-02722-3)
Supplement: Supplementary file 1 — On the Mechanism of the NPM1 Mutation in Acute Myeloid Leukemia [file 41375_2025_2722_MOESM1_ESM.pdf]

On the Mechanism of NPM1 Mutations in Acute Myeloid Leukemia

Michael R. Lieber and Chih-Lin Hsieh

SUPPLEMENTARY TEXT

Discussion of the Detailed Aspects of the Apobec3G Model for NPM1 Mutations

For nucleotide (nt) additions of the type seen in 97.2% of NPM1 mutations with complete or partial duplication of the 5'-TCTG sequence, a single-strand break (SSB) is needed at a nearby position. It is important to distinguish the original TCTG *versus* the complete (TCTG) or partial (xxTG) duplicate of it immediately downstream (i.e., on its right) in 97.2% of the 2322 cases. The original TCTG at c.860-863 does not vary in any of the 2322 patient cases (1). The first T nt (c.860) in the TCTG sequence is required for coding Leu (L) as part of the nuclear export sequence (NES). The C nt at c.861 can retain the same Leu amino acid (aa) if the sequence varies, and this nt is not mutated in any of the 2322 patient cases. The same important point applies to the top (non-template) strand T and G at c.862 and c.863 in the original TCTG sequence, which could vary substantially and still satisfy the reduction in aromaticity of the nuclear export sequence; yet these nts do not vary in the original TCTG in any of the cases (1). Similarly, if the ssDNA break occurred on the *top strand*, any sequence change at c.859 in the resynthesis would not alter the amino acid sequence (Leu), and yet no variation at c.859 is observed among the 2322 NPM1 patients (1). Taken together, there is no indication that repair synthesis occurs involving the nts in the top strand of the original 5'-TCTG-3' sequence (c.860 to 863), or immediately upstream (to the left of the 5'-TCTG-3' original sequence). These features place an upstream boundary on the region of new synthesis. Given the 5'→3' directionality of DNA synthesis, the local DNA synthesis involved in copying the top strand TCTG sequence for the duplication must initiate from a SSB on the bottom strand.

Several additional points beyond those in Figure 1 are worth noting. Firstly, Apobec3 enzymes require a single-stranded DNA substrate, at least locally. Such ssDNA can arise during replicative DNA synthesis or during transcription as the transcription bubble moves through the region. Transcription also generates some degree of transient single-strandedness in the wake (upstream trailing region) of the RNA polymerase due to transient negative supercoiling of the DNA duplex (2). This is illustrated by the increase in the local action of activation-induced deaminase (AID), which is another Apobec family member (3-6).

Secondly, for the events in which the NPM1 mutation (i.e., duplicate repeat) varies from the TCTG sequence, these variations are consistently in the 4-nt repeat on the right side (downstream in a transcription direction) rather than in the original 4-nt TCTG (upstream, or left side). Pol beta has a low but significant strand displacement capability (7). The known misincorporation propensities documented for human pol beta correspond very well with the single base mismatches in the subset of NPM1 4-nt duplications that have a single deviation in the second repeat (8, 9). Moreover, pol beta misincorporation in short gap repair of this type is known to have a very high incidence of two adjacent misincorporations that would explain the 10% of events with 2 adjacent mismatches within the 4-nt duplication (8, 9).

Importantly, many of the much less frequent NPM1 mutations (8 or fewer events among 2322 AML cases) may be explained by Apobec3G events initiating from other CC positions on the bottom strand (i.e., c.869 or c.872).

Thirdly, recent data shows Apobec3G can bind to ssDNA via its non-catalytic domain (CD1) in regions with abundant AA motifs to help position its catalytic domain (CD2) to its CC target sites (10). The AA motifs can be in the ssDNA (during strand separation of DNA synthesis) or in the RNA during transcription (via RNA tethering) (3-6). The predominant form of Apobec3G is dimeric. Therefore, binding of one of the CD1 domains to AA would tether two CD2 catalytic regions to the nearby DNA. Relevant to this, abundant consecutive AA sites are present immediately upstream and downstream of the duplicated region in the NPM1 gene. All these aspects would be consistent with the involvement of Apobec3G.

SUPPLEMENTARY FIGURE and LEGEND

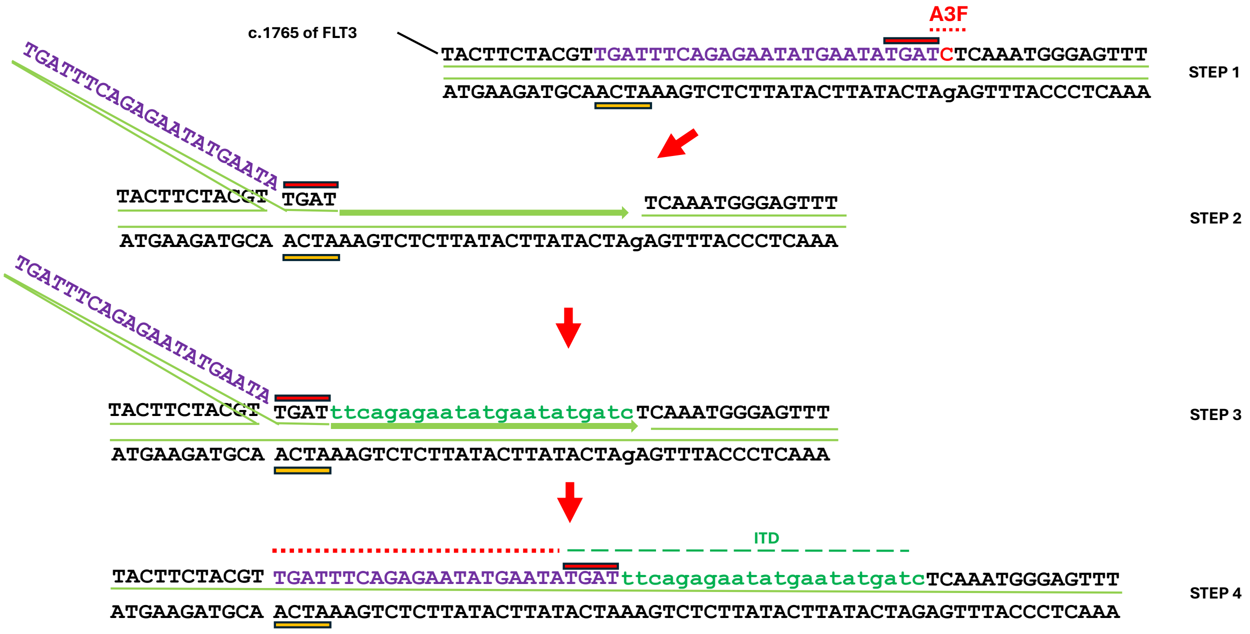

Supplementary Figure 1

Supplementary Figure 1. Possible Mechanism for Some of the Recurrent FLT3 ITD Events.

The FLT3 gene mutations are much more diverse than NPM1 mutations. However, some of the events are recurrent across many unrelated patients (11). A possible Apobec3 mechanism for one of the two recurrent Internal Tandem Duplications (ITD) with the *most frequent start position* is considered here.

STEP 1 The FLT3 sequence shown is the site of a tandem duplication beginning at the 5' TGAT 3' marked by the red bar above the top DNA strand (11). *The adjacent red C is within the Apobec3F (A3F) preferred deamination site (5' TCT 3'), which is noted with a dashed red line and red A3F label above it*. Deamination of this red C in the same biochemical steps detailed in the Figure 1 (Lines 1 to 3) would generate a one base gap.

In STEP 2, breathing during repair or replication (sequence in **purple**, which is AT-rich), followed by slippage of the red bar TGAT to the TGAT sequence located 21 nt upstream (underlined by **orange** bar under the bottom DNA strand) is illustrated.

In STEP 3, DNA polymerase extension (sequence in green and thick green horizontal arrow) generating an internal tandem duplication (ITD) shown in lower case letters is followed by ligation.

In STEP 4, separation of the top and bottom strands during S phase would permit duplication of the top strand to incorporate this specific recurrent FLT3 ITD outcome (one of the Type A event outcomes summarized by Borrow et al) (11). The **purple** sequence with the dashed red line above the top strand is the original sequence (same DNA as the upper case purple sequence in STEP 1). The dashed green line above the bright green lower case sequence is the ITD.

Minor mechanistic variations of this FLT3 ITD could lead to some of the other sequence variations assembled in the excellent comprehensive Borrow et al study of FLT3 ITD (11). For example, DNA polymerase addition at the initial Apobec3F gap could lead to the FLT3 ITD Type D event (which has no duplication, but merely an addition that included misincorporations). FLT3 ITD Type G events would involve a second slippage before the top strand is ligated, and this would result in triplication rather than duplication. FLT3 ITD Type B and C events could involve DNA polymerase extension beyond the initial Apobec3F single nt gap site. As mentioned, the error rate for misincorporation by pol beta can increase significantly during fill-in synthesis longer than a single nt (8, 9). (Participation of other error-prone polymerases to generate misincorporations or untemplated additions is also quite possible.) In the diagram, the first nucleotide on the far left is c.1765 in the coding DNA sequence, consistent with the Borrow et al. numbering (11).

SUPPLEMENT REFERENCES

1. Borrow J, Dyer SA, Akiki S, Griffiths MJ. Molecular roulette: nucleophosmin mutations in AML are orchestrated through N-nucleotide addition by TdT. Blood. 2019;134:2291-2303.

2. Sinden RR. DNA Structure and Function. San Diego: Academic Press; 1994:398.

3. Liu D, Loh YE, Hsieh CL, Lieber MR. Mechanistic basis for chromosomal translocations at the E2A gene and its broader relevance to human B cell malignancies. Cell Rep. 2021;36:109387.

4. Liu D, Lieber MR. The mechanisms of human lymphoid chromosomal translocations and their medical relevance. Crit Rev Biochem Mol Biol. 2022;57:227-243.

5. Liu D, Goodman MF, Pham P, Yu K, Hsieh CL, Lieber MR. The mRNA tether model for activation-induced deaminase and its relevance for Ig somatic hypermutation and class switch recombination. DNA Repair (Amst). 2022;110:103271.

6. Liu D, Hsieh CL, Lieber MR. The RNA tether model for human chromosomal translocation fragile zones. Trends Biochem Sci. 2024;49:391-400.

7. Hartenstine MJ, Goodman MF, Petruska J. Weak strand displacement activity enables human DNA polymerase beta to expand CAG/CTG triplet repeats at strand breaks. J Biol Chem. 2002;277:41379-41389.

8. Osheroff WP, Jung HK, Beard WA, Wilson SH, Kunkel TA. The fidelity of DNA polymerase beta during distributive and processive DNA synthesis. J Biol Chem. 1999;274:3642-3650.

9. Kunkel TA. Considering the cancer consequences of altered DNA polymerase function. Cancer Cell. 2003;3:105-110.

10. Yang H, Pacheco J, Kim K, Bokani A, Ito F, Ebrahimi D et al. Molecular mechanism for regulating APOBEC3G DNA editing function by the non-catalytic domain. Nat Commun. 2024;15:8773.

11. Borrow J, Dyer SA, Akiki S, Griffiths MJ. Terminal deoxynucleotidyl transferase promotes acute myeloid leukemia by priming FLT3-ITD replication slippage. Blood. 2019;134:2281-2290.
